# Differentially Expressed Proteins in Chronic Active Hepatitis, Cirrhosis, and HCC Related to HCV Infection in Comparison With HBV Infection: A proteomics study

**DOI:** 10.5812/hepatmon.8351

**Published:** 2013-07-03

**Authors:** Jamal Sarvari, Zahra Mojtahedi, Seyed Ali Reza Taghavi, Yasuhiro Kuramitsu, Mahmoud Shamsi Shahrabadi, Abbas Ghaderi, Kazuyuki Nakamura

**Affiliations:** 1 Institute for Cancer Research, Shiraz University of Medical Sciences, Shiraz, IR Iran; 2 Department of Bacteriology and Virology, Shiraz University of Medical Sciences, Shiraz, IR Iran; 3 Department of Internal Medicine, Shiraz University of Medical Sciences, Shiraz, IR Iran; 4 Department of Virology, Tehran University of Medical Sciences, Tehran, IR Iran; 5 Department of Biochemistry and Functional Proteomics, Yamaguchi University Graduate School of Medicine, Yamaguchi, Japan

**Keywords:** Cirrhosis, Hepatitis B Virus, Hepatitis C Virus, Hepatocellular Carcinoma

## Abstract

**Background:**

Hepatocellular carcinoma is a highly progressive cancer in the case of late diagnosis which is frequently associated with HBV and HCV viral infections.

**Objectives:**

To identify differentially expressed serum proteins among three main stages of HCV infection and healthy individuals, and their comparisons with sera from patients with the same stage of HBV infection.

**Patients and Methods:**

Two-dimensional polyacrylamide gel electrophoresis combined with liquid chromatography-tandem mass spectrometry was performed on 47 sera from healthy volunteers, those with chronic active hepatitis, cirrhosis and HCC patients associated with HBV and HCV infections.

**Results:**

Among these, 62 spots were differentially expressed (≥ 1.5 fold; P < 0.05), of which 42 spots that corresponded to 15 proteins were identified by liquid chromatography-tandem mass spectrometry. CD5-like antigen (CD5L) was differentially expressed between cirrhosis and HCC patients with HCV infection. Leucine-rich α2-glycoprotein (LRG) and haptoglobin (HP) α2 isoforms differed in the HCC that was associated with either HCV or HBV infections.

**Conclusions:**

CD5L might be a useful biomarker for early diagnosis of HCC in HCV cirrhotic patients. LRG and HP α2 isoforms could be potential markers for distinguishing viral HCC. Our results also further support the presence of varying molecules involved in hepatocarcinogenesis in HBV when compared with HCV infection.

## 1. Background

Hepatocellular carcinoma (HCC) is the seventh most frequent cancer and the third cause of mortality from cancer worldwide (1). Its major risk factors are hepatitis B virus (HBV), hepatitis C virus (HCV), aflatoxin B1 exposure, and alcohol consumption (1, 2). Approximately 350 million individuals are infected with HBV which is the underlying reason of 50% of HCC cases, and 170 million are infected with HCV which is the cause of 30% of HCC cases (3, 4). About 20%-30% of patients usually develop liver cirrhosis, from which 80%-90% of HCC cases arise (1). HCC occurs at an annual rate of 1%-7% in HCV-infected cirrhotic patients, and 3%-8% in HBV-infected cirrhotic patients (5, 6). Therefore, HCC is considered a predictable cancer for which screening is generally recommended in high-risk groups such as cirrhotic patients (7). Alpha-Fetoprotein is the most widely used serum biomarker for the detection of HCC, but has less reliability during the early stages of HCC (8). While surgical resection or liver transplant remains the most effective options for curing HCC, the majority of cases are not candidates for surgery because of their interahepatic or distant metastasis at the time of diagnosis (9). More investigations to find useful biomarkers for early diagnosis and elucidative mechanisms of hepatocarcinogenesis as new therapeutic targets are urgently needed for HCC. There are substantial differences between the mechanisms of HBV and HCV in induction of malignancy. Besides the oncogenic potential of viral proteins, HBV is a DNA virus able to integrate into the host DNA, directly triggering and transforming hepatocytes. In contrast, HCV (an RNA virus) is unable to integrate into the host genome, but it seems that viral proteins have more significant roles in hepatocarcinogenesis (10). Genomic studies on liver tissues have shown inconsistent gene expression profiles between HCC related to HBV and the one related to HCV (11, 12). Proteomic analysis of the liver tissues has also revealed different protein profiles between HBV and HCV-infected patients (13). Although biomarker studies on liver tissues could be a useful strategy for determining new pathogenic biomarkers (for diagnostic and/or prognostic processes), serum has much priority for finding inexpensive, easily applicable, noninvasive biomarkers. Two-dimensional polyacrylamide gel electrophoresis (2-DE), in which comparisons can be made between normal and/or diseased samples, has a powerful capacity for separating thousands of proteins, including tissues and body fluids. This technique followed by protein identification with mass spectrometry has opened a new window for the discovery of biomarkers (2). By employing comparative proteomic approaches several putative serum HCC biomarkers have successfully been identified; such as heat shock protein27, C3, Apolipoprotein AI, haptoglobin (HP), α-1-antitrypsin (AAT) and transthyretin (TTR) in HBV-infected patients and apolipoprotein C I and II, α-anolase, transferrin, and galectin-4 in HCV-infected cases (2, 8, 11, 13).

## 2. Objectives

We studied serum biomarkers during three stages in HBV-infected patients [chronic active hepatitis (CAH), cirrhosis, and HCC] and healthy individuals (14). In the present study, we have decided to:

a) Investigate serum proteomes among patients in the three stages of HCV infection and healthy individuals.

b) Compare the three stages of HCV infection with those in the same stage of HBV infection by using 2-DE coupled to liquid chromatography-tandem mass spectrometry. To the best of our knowledge, information on serum proteome profiles of HCC related to HBV and HCV is very limited. Identification of these differentially expressed proteins may provide possible specific serum biomarkers for the early diagnosis of HCC related to these hepatotropic viruses, and/or provide information for clarifying mechanisms of liver carcinogenesis related to these viruses.

## 3. Patients and Methods

### 3.1. Subjects

Patients (N = 40) were recruited consecutively from the Departments of Gastroenterohepatology and Organ Transplant Surgery, Nemazie Hospital, Shiraz, Iran from September 2007 to July 2009. Characteristics of the study groups were obtained from patients’ files, as shown in Tables 1 and 2. 

**Table 1.** Clinical and Laboratory Characteristics of the Study Groups

Characteristics	Healthy (n=7)	CAH^a^-HBV (n=7)	C-HBV (n=7)	HBV-HCC (n=7)	CAH-HCV (n=7)	C-HCV (n=7)	HCV-HCC (n=5)
**Sex**							
Male	6	5	6	6	6	5	4
Female	1	2	1	1	1	2	1
**Age** **, y**							
(mean±SD^a^)	42±7	42±8	40±7	43±11	42±11	54±10	39±12
**HBsAg^a^**	-	+	+	+	-	-	-
**HBV DNA**		+	+	+	-	-	-
**HCVAb**	-	-	-	-	+	+	+
**HCV RNA**	-	-	-	-	+	+	+

^a^Abbreviations: SD, standard deviation; HBsAg, hepatitis B surface antigen; CAH, chronic active hepatitis

**Table 2.** Clinical and Laboratory Characteristics of 12 HCC Patients

No.	Age, y	Gender	HBeAb/HBeAg	HBcAb/HCVAb	HBV/HCV genotype	AFP^a^	ALT^a^	AST^a^	Cirrhosis	Child-pugh
**1**	32	M	+/+	+/-	D	10.2	55	67	+	B
**2**	53	M	+/-	+/-	D	11.4	134	163	-	B
**3**	44	F	+/-	+/-	D	14.1	107	146	-	A
**4**	53	M	+/-	+/-	D	653	39	138	+	C
**5**	51	M	+/+	+/-	D	724	107	166	+	B
**6**	53	M	+/-	+/-	D	96.3	394	365	+	B
**7**	54	M	+/-	+/-	D	1.3	37	22	-	C
**8**	55	M	-/-	-/+	Serum HCV RNA was negative	15	43	127	+	C
**9**	54	M	-/-	-/+	3a	6.2	52	80	-	B
**10**	46	M	-/-	-/+	3a	6	75	146	+	B
**11**	47	M	-/-	-/+	1a	5	105	144	-	C
**12**	32	M	-/-	-/+	Not determined	8	26	28	+	B

^a^ Abbreviations: AFP, alpha-feto protein; ALT, Alanine aminoteransferase; AST, Aspartate aminoteransferase

There were 19 HCV-positive patients that included 7 with CAH, 7 with cirrhosis, and 5 with HCC. A total of 21 patients were HBV-positive of which 7 were diagnosed with CAH, 7 with cirrhosis, and 7 with HCC. Their diseases were confirmed by biochemical, virological, imaging, and pathological examinations. Included in this study were 7 age and sex matched healthy individuals with no histories of liver diseases, HBV and HCV laboratory signs, malignancies, and recent or chronic infectious diseases. Written informed consent was obtained from each participant before sampling. The Study was approved by the ethic committee of Shiraz University of Medical Sciences.

### 3.2. Serum Samples

A 5 mL blood sample was drawn from each participant and allowed to clot for 2 h. Blood samples were spun at 3000 rpm for 10 min and the serum was separated, aliquoted, and stored at -70°C until tested. In order to increase serum protein resolution, high abundant proteins that included albumin and immunoglobulin (Ig) G were depleted from 60 µL of serum by the Arum Protein Mini Kit (Bio Rad, Hercules, CA, USA). Subsequently, protein concentration of the depleted sera was determined by a Bradford protein assay, using albumin as the standard.

### 3.3. Laboratory Tests

HBV and HCV genotyping was performed using polymerase chain reaction-restriction fragment length polymorphisms and genotype specific primers respectively, as previously described (15, 16). α-fetoprotein was detected by commercial quantities Enzyme linked Immunosorbent Assay kits (ConAg, Sweden) according to the manufactures' instructions.

### 3.4. 2-DE

Briefly, approximately 100 µg of proteins were loaded into immobilized pH gradient strips pH 3-10 linear (Bio-Rad, Hercules, CA, USA) in first dimensional isoelectric focusing. The rehydration solution contained 8 M urea, 3% CHAPS, 2% immobilized pH gradient buffer (pH 3-10), 50 mM Dithiothreitol and a trace amount of bromophenol blue. The strips were focused at 80000 Vh. The focused strips were equilibrated and reduced to 10 mL equilibration buffer [50 mM Tris (pH 8.8), 6 M urea, 30% (w/v) glycerol, 2% (w/v) sodium dodecyl sulfate] that contained 1% (w/v) Dithiothreitol for 15 min and alkylated in another 10 mL equilibration buffer that contained 2.5% (w/v) idoacetamide for 15 min. The strips were sealed on top of a 12.5% sodium dodecyl sulfate gel using 0.5% agarose. The second dimensional electrophoresis was performed in the protean II xi cell (Bio-Rad). Electrophoresis was run at 10 mA per gel for 30 min followed by 25 mA per gel until the tracking dye reached the bottom of the gels. The gels were visualized by using a complete protocol of a silver staining method for analytical gels. For preparative gels, the method was modified to make the standard protocol compatible with mass spectrometry analysis (17).

The silver-stained gels were scanned using a GS-800 calibrated densitometer (Bio-Rad) at 300 dpi. Gel images were analyzed by Prognosis software (Nonlinear, Newcastle-upon-Tyne, UK) according to the instruction procedure for differentially expressed proteins. The protein spots whose normalized volumes changed more than 1.5 fold and with P < 0.05 were picked up from the gels that had been stained with the mass spectrometry compatible method.

### 3.5. In-gel Digestion and Liquid Chromatography-Tandem Mass Spectrometry Analysis

In-gel digestion was carried out as previosly described (18). For liquid chromatography-tandem mass spectrometry analysis, the lyophilized samples were resuspended in 0.1% formic acid before analysis. An Agilent 1100 LC/MSD trap XCT was used for high-performance liquid chromatography and tandem mass spectrometry. The mobile phase A of liquid chromatography was water/0.1% formic acid and the mobile phase B was acetonitril/0.1% formic acid. A trap column (Agilent, G 1375-87320, 105 mm, 25 µm, Germany) was connected to a standard column (Zobrax 300 SB-C18, 75 mm, 3.5 µm). Twelve µL of the peptide was loaded on a trapping column and desalted by washing with 2% B for 5 min. A linear gradient from 2%-60 % of concentration B in 55 min, then 80% B in 8 min, and re-equilibration of 2% B in 10 min, was applied to elute peptides at a flow rate of 300 nL/min. The mass spectrometer was operated in positive ion mode over the range of 350-1850 m/z. Tandem mass spectrometry data were analyzed with spectrum mill (Agilent, Palo Alto, CA, USA) against the Swiss-Prot database (released May, 2010). The following filters were used after database searching: peptide score 8, peptide % SPI > 70 and protein score 10.

## 4. Results

**Figure 1.**
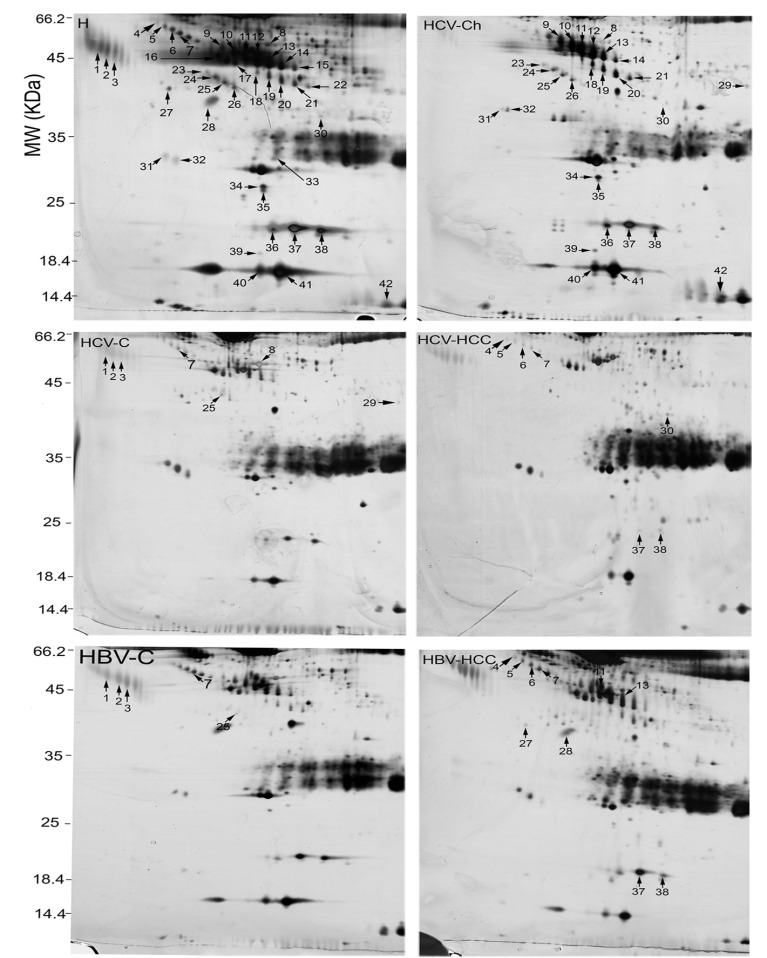
Identified Differentially Expressed Spots Among 2-DE Gels of the Sera From Healthy Individuals (H), Chronic Active Hepatitis (Ch), Cirrhosis (C), and HCC Associated With HCV and HBV Infections Were Numbered. Spot numbers are for cross-reference with Table 3 and Figures 2-4.

In the present study, serum proteome analysis was carried out for 7 healthy individuals and 19 HCV-positive patients in three stages (7 with CAH, 7 with cirrhosis, and 5 with HCC). Serum proteomes of the 19 HCV-positive patients were further compared to those from 21 HBV-positive patients (7 with CAH, 7 with cirrhosis, and 7 with HCC) who were of the same stage. Among these groups, 62 protein spots were differentially expressed (≥ 1.5 fold; P <0.05; Table 3). Of these, 42 spots were identified by MS. The identified spots corresponded with 15 proteins. Table 3 displays the molecular weights (MW), pI, accession numbers, and proposal function of the identified proteins. The locations of these spots on the gels are shown in Figure 1.

**Table 3.** List of 42 Protein Spots Identified By Liquid Chromatography-Tandem Mass Spectrometry. Spot Numbers are Related to Figures 1-4

Spot No	Protein name	Mr ^a^ (Pre ^a^/Exp ^a^)	PI ^a^ (Pre/Exp)	Acc ^a^ No.	Coverage, %	Function
**1**	α-1 acid glycoprotein	23.5/44	4.93/4	P02763	8	Immune system
**2**	α-1 acid glycoprotein	23.5/43	4.93/4.7	P02763	8	Immune system
**3**	α-1 acid glycoprotein	23.5/43	4.93/4.7	P02763	8	Immune system
**4**	Leucine-rich α-2-glycoprotein	38.1/48	6.45/4.3	P02750	3	Unknown
**5**	Leucine-rich α-2-glycoprotein	38.1/47	6.45/4.4	P02750	38	Unknown
**6**	Leucine-rich α-2-glycoprotein	38.1/46	6.45/4.6	P02750	24	Unknown
**7**	Leucine-rich α-2-glycoprotein	38.1/45	6.45/4.7	P02750	10	Unknown
**8**	CD5-like antigen	38/40.5	5.28/5.85	O43866	22	Immune system
**9**	Haptoglobin-α chain	45.2/42	6.13/5.3	P00738	7	Hemoglobin scavenger
**10**	Haptoglobin-α chain	45.2/41	6.13/5.4	P00738	24	Hemoglobin scavenger
**11**	Haptoglobin-α chain	45.2/39	6.13/5.6	P00738	14	Hemoglobin scavenger
**12**	Haptoglobin-α chain	45.2/38	6.13/5.75	P00738	30	Hemoglobin scavenger
**13**	Haptoglobin-α chain	45.2/37.5	6.13/5.85	P00738	4	Hemoglobin scavenger
**14**	Haptoglobin-α chain	45.2/37	6.13/6	P00738	10	Hemoglobin scavenger
**15**	Haptoglobin-α chain	45.2/35.5	6.13/6.2	P00738	30	Hemoglobin scavenger
**18**	Haptoglobin cleaved-α chain	45.2/36	6.13/5.7	P00738	23	Hemoglobin scavenger
**19**	Haptoglobin cleaved-α chain	45.2/35	6.13/5.85	P00738	8	Hemoglobin scavenger
**20**	Haptoglobin cleaved-α chain	45.2/34	6.13/6.1	P00738	38	Hemoglobin scavenger
**21**	Haptoglobin cleaved-α chain	45.2/33.5	6.13/6.2	P00738	33	Hemoglobin scavenger
**22**	Haptoglobin cleaved-α chain	45.2/33	6.13/6.25	P00738	6	Hemoglobin scavenger
**36**	Haptoglobin-α2 chain	45.2/17	6.13/5.9	P00738	21	Hemoglobin scavenger
**37**	Haptoglobin-α2 chain	45.2/17	6.13/6.2	P00738	16	Hemoglobin scavenger
**38**	Haptoglobin-α2 chain	45.2/17	6.13/6.6	P00738	25	Hemoglobin scavenger
**16**	Complement C3	187.1/39	6.02/5.05	P01024	16	Innate immunity
**17**	Zinc-α-2-glycoprotein	33.8/38	5.57/5.3	P25311	4	Stimulate lipolysis
**23**	Clusterin	52.4/36	5.89/5.1	P10909	5	Unknown
**24**	Clusterin	52.4/35	5.89/5.2	P10909	32	Unknown
**25**	Clusterin	52.4/34	5.89/5.3	P10909	5	Unknown
**26**	Clusterin	52.4/33	5.89/5.4	P10909	24	Unknown
**27**	α-1-antitrypsin	46.7/33	5.37/4.7	P01009	13	Protease inhibitor
**28**	Transthyretin	15.8/33	5.52/5.2	P02766	54	Thyroxine hormone carrier
**40**	Transthyretin	15.8/14.4	5.52/5.8	P02766	15	Thyroxine hormone carrier
**41**	Transthyretin	15.8/14.4	5.52/6.1	P02766	41	Thyroxine hormone carrier
**29**	Immunoglobulin gamma-2-chain C	35.9/35	7.66/7.5	P01859	24	Adaptive immunity
**30**	Ficolin 3	32.9/31	6.2/6.75	O75636	8	Innate immunity
**31**	Immunoglobulin J chain	15.5/23	4.62/4.4	P01591	44	IgA and IgM linker protein
**32**	Immunoglobulin J chain	15.5/23	4.62/4.6	P01591	31	IgA and IgM linker protein
**33**	Immunoglobulin kappa chain	11.6/24	5.58/5.65	P01838	33	Adaptive immunity
**34**	Retinol-binding protein	23/21.5	5.76/5.8	P02753	6	Carrier
**35**	Retinol-binding protein	23/21	5.75/5.8	P02753	29	Carrier
**39**	Albumin	69.3/16	5.92/5.8	P02768	8	Carrier
**42**	Hemoglobin subunit delta	16/13	7.84/7.8	P02042	9	Oxygen transport

^a^ Abbreviations: Pre, Predicated; Exp, experimental; PI, Isoelectric point; Acc, Accession numbers; Mr, Relative molecular mass

There was no differentially expressed protein spots between CAH related to HBV and CAH related to HCV infections. However, comparisons of other stages revealed several differential protein spots as below.

### 4.1. Healthy Individuals Versus HCV-Positive Patients

Differentially expressed proteins among these four groups were HP α and α2 isoforms, clusterin (CLU), retinol binding protein, TTR, albumin, α1-acid glycoprotein (AGP), hemoglobin delta, Ig J chain, Ig gamma chain c, C3, zinc-α glycoprotein, and CD5-like antigen (CD5L) (Table 3). 

### 4.2. CAH-HCV Patients Versus Cirrhosis-HCV Patients

Analysis of serum protein expressions between CAH and cirrhosis in HCV-positive patients revealed 35 differentially expressed protein spots. From these, 25 were identified by MS. HP α and α-2 isoforms, CLU, retinol binding protein, TTR, albumin, AGP and hemoglobin delta were decreased in the cirrhosis stage but IgJ chain was increased.

### 4.3. Cirrhosis-HCV Patients Versus HCC-HCV Patients

**Figure 2.**
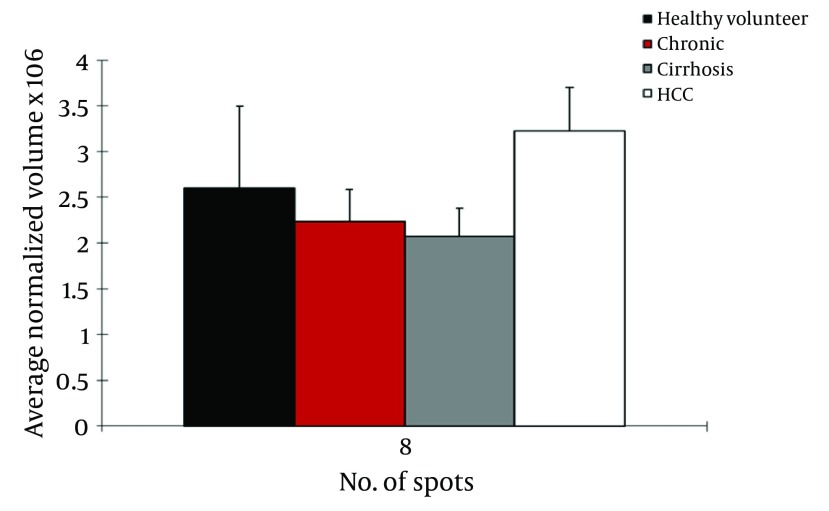
Normalized Volume Intensity of CD5-Like Antigen (Spot no. 8) in the Sera From Healthy Volunteers, CAH, Cirrhosis, and HCC Patients Related to HCV

We observed differential expression of 13 protein spots between cirrhosis and HCC patients with HCV infection. From these, 2 protein spots were identified by MS. CD5L (> 1.6fold; P = 0.022) was up regulated in HCC patients but Ig gamma chain C (> 3.4 fold; P < 0.042) was down regulated in this group (Figure 2). 

### 4.4. Cirrhosis-HBV Patients Versus Cirrhosis-HCV Patients

**Figure 3.**
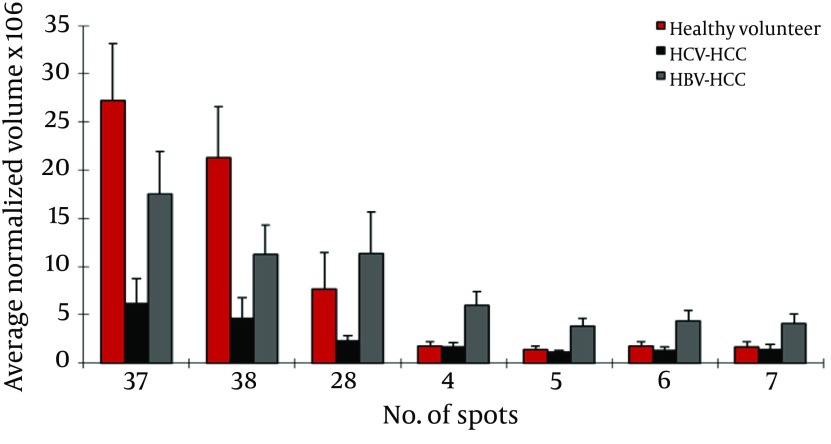
Normalized Volume Intensity of Haptoglobin-α Isoforms (Spots No. 37, 38), Transthyretin (Spot No. 28) and Leucine Rich α2-Glycoprotein (Spots No. 4-7) Between HCC Associated With HBV and HCV Patients.

Of the seven differentially expressed protein spots between the two groups, six were identified by MS. In HBV patients with cirrhosis, AGP was up regulated in three spots; also, CLU and leucine-rich α-2-glycoprotein (LRG) were up regulated and Ig gamma chain C was down regulated in this group compared to HCV cirrhosis (Figure 3). 

### 4.5. HCC-HBV Patients Versus HCC-HCV Patients

**Figure 4.**
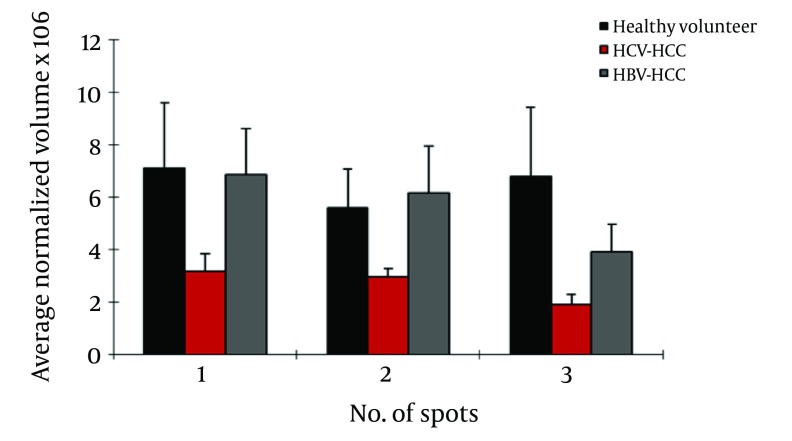
Normalized Volume Intensity of α1-Acid Glycoprotein (Spots no. 1-3) Between Patients With Cirrhosis Related to HBV and HCV.

This analysis showed 21 protein spots differentially expressed between these groups. Of them, 11 were identified by MS as belonging to 5 different proteins. LRG (4 spots), HP α-2 isoforms (2 spots), HP- α isoforms (2 spots), TTR, and AAT were up regulated in HBV-HCC patients compared to HCV-HCC patients; but ficolin-3 was down regulated in this group (Figure 4). 

## 5. Discussion

Advance in proteomic and genomic studies in recent years have highlighted heterogeneity in pathogenesis of HCC. A number of proteins and genes have been identified with differentially expression in HCV- and HBV-infected patients with liver pathology (10). We investigated the serum proteomes in individuals with three stages of HCV infection (CAH, cirrhosis, and HCC) and healthy individuals by 2-DE coupled to mass spectrometry and compared them to those of similar stages in HBV infection. In the HCV-infected patients, we found increased CD5L expression at the HCC stage compared to cirrhosis. An increased serum level of CD5L has been reported in HCV-cirrhotic compared to mild fibrosis patients by Gangadharan et al. (19), in cirrhotic and HCC patients compared to pre-cirrhotic disease related to non-alcoholic fatty liver disease by Gray et al. (20), and in Kawasaki disease and atopic dermatitis (20-22). Increased expression of CD5L mRNA has also been reported in cirrhotic and cancerous liver tissues (20). Our report is the first one to show over-expression of CD5L in HCV-HCC compared to HCV-cirrhosis. CD5L is a secreted glycoprotein belonging to the scavenger receptor cysteine-rich super-family. It is expressed by macrophages present in both primary and secondary lymphoid tissues including the thymus, bone marrow, spleen, and lymph nodes. CD5L binds to myelomonocytic and lymphoid cells, suggesting that it may play a role in the regulation of both the innate and adaptive immune systems (23). Evidence suggests an association of CD5L with IgM (24), an antibody that is a cost-effective serological marker of active viral replication in HCV-infected patients (23-25). The exact function of CD5L is not clear. Recently, Haruta et al. have shown that CD5L supports macrophage survival and activity in Corynebacterium parvum-induced hepatitis in mice (21). Due to the association of CD5L with the immune system, an increase in expression of CD5L in HCV-HCC patients compared to those with cirrhosis may reflect the nature of an immune response to HCV infection. Simultaneously, it may be considered a variation in the oncogenic behavior of HCV in HCC patients compared to cirrhotic patients with HCV. We have previously investigated differentially expressed serum proteins among CAH, cirrhosis, and HCC stages of HBV infection and healthy individuals by 2-DE coupled to mass spectrometry (14). Here we compared the serum proteomes of these stages of HCV infection versus HBV infection. These comparisons revealed different expressions of several key proteins, notably LRG, HP α-2 isoforms, TTR, and AGP. LRG was one of the serum-overexpressed proteins in HBV-HCC compared to HCV-HCC patients. LRG down regulation has been reported in HCV-cirrhotic patients when compared to mild fibrosis (26). It has been reported that the serum level of this protein has increased in malignancies such as lung and ovarian cancer, as well as bacterial and viral infections (27). LRG was first isolated from serum (28). Although liver cells appear to be the major source of this protein, it is also produced by mature neutrophils and endothelial venules of the mesenteric tissues (27). Its real physiological functions have not yet been clarified. LGR is induced by interleukin-6, interleukin-1-beta and transforming growth factor-alpha in hepatoma cells, and is over-expressed in livers of the mice challenged by lipopolysaccharide, rendering it an acute phase protein. With the current knowledge, the reason for LRG level differences between HBV-HCC and HCV-HCC is not clear. LRG is suggested to be a marker for poor prognosis in HCC (29). HP is a tetramer molecule composed of two á subunits (9.1 kDa) and two β subunits (40 kDa). Although the liver is the major source of serum HP, it is also secreted by some cancer cells (30). HP is a positive acute phase protein and has long been used for the study of various liver diseases such as viral hepatitis and HCC (31). We have identified 15 spots as HP, 3 of which were HP-α2 and the remainders were of the HP-α chain. Only two spots of the HP-α2 chain were differentially expressed between HBV-HCC and HCV-HCC patients. We have previously observed the differential expression of some HP isoforms between HBV-HCC and HBV-cirrhosis (14). In keeping with our data, specific HP profiles have been reported in breast, ovarian, head and neck cancers (32). Different HP isoforms may show different biological functions, and their occurrences might be associated with disease-specific alterations in the intercellular processes such as post-translational modification mechanisms. In this regard, Ang et al. have reported that HPs with different degrees of glycosylation are produced by HCC tissue, while other HP glycoforms are produced by normal cells (30). We have found 3 spots as TTR, one of which was of multimeric form. The multimeric form of the serum TTR increased (4.9 fold) in HBV-HCC patients compared to HCV-HCC patients. Also, two of the monomeric forms were down regulated (5.6 fold) in the cirrhosis patients sera compared to CAH associated with HBV. TTR (also called prealbumin) is presented in the serum and cerebrospinal fluid that has been synthesized and secreted by liver cells and the choroid plexus of the brain. The two significant physiological functions of TTR are transport of thyroxin (T4) and retinol (vitamin A) (33). Considering the fact that the liver is the source of serum TTR, it is reasonable to assume that the synthesis of this protein varies in liver diseases such as cancer and hepatitis. The mRNA level of this protein was decreased after treatment of HepG2 cells with interleukin-6, interleukin-1, or transforming growth factor alpha (34). TTR can inhibit interleukin-1 production by monocytes and endothelial cells, thus showing anti-inflammatory properties (35). Our finding in the 2-DE analysis has suggested conformational changes of TTR in HCC patients related to HBV and HCV. Differential expressions of TTR in the sera have been reported in SARS, dengue fever, ovarian cancer, malignant melanoma, and in the cerebrospinal fluid of some neurological disorders such as Alzheimer's, Parkinson, and schizophrenia (34, 36, 37). We found down regulation of AGP in HCV-cirrhotic patients compared to those with HBV. AGP is an acute phase serum glycoprotein synthesized and secreted by the liver. AGP is an indicator of liver failure after liver resection (38). A change in concentration and glycosylation of AGP is known to be related to the pathogenesis of liver diseases. It increases in patients with acute hepatitis and HCC, but decreases in patients with chronic hepatitis and liver cirrhosis (39). Since AGP is synthesized and secreted by hepatocytes, damage and injury to liver parenchyma can affect the serum concentration of this protein. Decreased expression of AGP in HCV-cirrhotic patients results in massive liver tissue damage in HCV compared to HBV cirrhotic patients that may be associated with different hepatopathogenesis mechanisms induced by these hepatotropic viruses. Although we have identified several differentially expressed proteins among different stages of HCV infection and compared them to those in different stages of HBV infection, some limitations still exist. The identified proteins should be confirmed by other techniques such as western blotting, real-time PCR or ELISA in a larger number of the patients. In conclusion, differentially expressed proteins, e.g. CD5L, in the sera from CAH, cirrhosis, and HCC related to HCV were identified using a proteomic approach. We have also compared, for the first time, the serum proteomes of these three main stages of HCV infection with the same stages of HBV infection and identified some relevant differentially expressed proteins such as LRG and HP α2 isoforms. Further studies are required to confirm the differential expression of the identified proteins and their significance as disease biomarkers.
